# Morphological and Phylogenetic Characterization of *Alternaria* Section *Undifilum* Fungal Endophytes from *Astragalus* and *Swainsona* spp.

**DOI:** 10.3390/jof11070541

**Published:** 2025-07-19

**Authors:** Marwa Neyaz, Olabisi Adebisi, Daniel Cook, Rebecca Creamer

**Affiliations:** 1 Entomology, Plant Pathology, and Weed Science, New Mexico State University, Las Cruces, NM 88003, USA; marwane@nmsu.edu; 2 Molecular Biology and Life Sciences, New Mexico State University, Las Cruces, NM 88003, USA; gadebisi@nmsu.edu; 3 USDA ARS Poisonous Plant Research Laboratory, 1150 E 1400 N, Logan, UT 84321, USA; daniel.cook@usada.gov

**Keywords:** *Astragalus*, *Swainsona*, *Alternaria*, fungal characterization

## Abstract

The locoweeds *Astragalus* and *Oxytropis* in the Americas and China, as well as *Swainsona* in Australia, harbor swainsonine-producing endophytes responsible for “locoism” or “pea struck” syndromes in grazing animals. Demonstration of *Alternaria* section *Undifilum* spp. requires demonstration of morphological characters such as a wavy germ tube and slow growth. While *Astragalus wetherilli*, *A. pubentissimus*, *Swainsona canescens*, and *S. galegifolia* plants have been shown to contain swainsonine, and fungi isolated from the plants have been partially characterized genetically, the fungi have not been characterized morphologically. This work sought to complete morphological characterization and determine species for those fungi and from fungi associated with *Swainsona luteola* and *S. brachycarpa.* The fungi were isolated from their hosts onto media and exhibited slow growth, resulting in a colony diameter of approximately 10 mm after 30 days. Morphological identification revealed production of conidia that produced a wavy germ tube for the endophytes from *Astragalus pubentissimus* species, *Swainsona canescens*, and *S. galegifolia*. Sequence analyses of the ITS region and the *swnK-KS* and *swnK-TR* genes of these fungi suggest that the fungi isolated from *Astragalus* are closely related and distinct from the fungi isolated from *Swainsona*. Presence of the *swnK* gene demonstrates that all the fungi have a necessary component to produce swainsonine. Fungi isolated from *Astragalus* spp. differed in color, growth, and conidium size, and/or their sequences. While the fungi isolated from *Swainsona canescens* and *S. galegifolia* endophytes differed in color, growth, and conidium size, those isolated from *Swainsona luteola* and *S. brachycarpa* did not produce conidia. Sequences from all *Swainsona* endophytes were almost identical and were concluded to be the same species. The new species described here are *Alternaria wetherii*, *A. pubentissima*, *A. pubentissimoides*, and *A. swainsonii.*

## 1. Introduction

Many species the legume genera of *Astragalus*, *Oxytropis*, and *Swainsona* have been demonstrated to be toxic to grazing animals. *Astragalus* and *Oxytropis* are endemic to the Americas and Asia, while *Swainsona* is a closely related genus within the Fabaceae family endemic to Australia [1]. Consumption of some species of *Swainsona* causes “pea struck” disease due to swainsonine content [2,3]. Similarly, consumption of toxic *Astragalus* and *Oxytropis* sp. locoweeds causes locoism due to swainsonine content [4,5,6]. Swainsonine is a secondary metabolite that inhibits α-mannosidase; sufficient consumption of plants containing swainsonine by grazing animals results in decreased mannosidase levels and dysfunctional glycoprotein processing [7,8,9]. Swainsonine was originally isolated from the *Swainsona canescens* in Australia [2] and was subsequently found in *Astragalus* and *Oxytropis* spp. [10,11]. Livestock that feed on plants containing swainsonine exhibit “locoism” or “pea struck” disease characterized by lack of muscular coordination, weight loss, altered behavior, depression, abortion, and eventually death, thus resulting in severe economic losses to the livestock industry [12].

Endophytic fungi isolated from *Astragalus* and *Oxytropis* in the western United States were found to produce swainsonine in media, which led to subsequent investigations into plant–fungal interactions and “locoism” disease [13]. All plants that contain swainsonine have been shown to contain fungi that produce the toxin, including *Swainsona* spp., and the morning glory *Ipomoea carnea* [14,15,16,17]. These isolates were initially characterized as possible species of *Alternaria* or *Embellisia* [13], and an isolate from *Oxytropis kansuensis* in China was described as *Embellisia oxytropis* [18]. Upon further morphological and molecular analyses, these endophytes were reported as conspecific members of a new genus in the Pleosporaceae, *Undifilum* [19]. *Undifilum* was subsequently reclassified as a section of *Alternaria* [20].

A limited number of fungal species are known to produce the toxic alkaloid swainsonine in different hosts. Swainsonine was shown to be produced by the fungus *Rhizoctonia leguminicola* [21], which was recently reclassified as *Slafractonia leguminicola* [22]. Further investigations demonstrated that the locoweeds contained endophytes that synthesize swainsonine [13]. Other fungi have been reported to produce swainsonine, including the insect pathogen *Metarhizium* spp., which affects insect hosts [23], and the dermatophytes of the Arthrodermataceae which affects mammal hosts [24].

*Alternaria* section *Undifilum* endophytes isolated from toxic legumes share key identification features. Conidia produced from these fungi are described as ovate with dark thick septa and present a distinctive wavy tube upon conidial germination [19]. Extreme slow growth of mycelia in media was also observed [13,17,19,25]. Molecular markers were utilized to determine the taxonomic placement of this group of endophytes including the internal transcribed spacer (ITS) and glyceraldehyde-3-phosphate dehydrogenase gene *gpd* [13,19,25,26,27,28]. Lawrence et al. [29] used *gpd* to differentiate *Alternaria* sp. into asexual sections. Baucom et al. [25] used ITS and *gpd* regions, as well as random amplified polymorphic DNA (RAPD) markers, and they showed that *Alternaria* species isolated from select *Astragalus* species formed a different clade from those isolated from *Oxytropis* species. Production of detectable levels of swainsonine was not a requirement for classification into *Alternaria* section *Undifilum*, and one member of the group, *Alternaria bornmuelleri*, does not produce detectable levels of swainsonine [19].

The swainsonine biosynthetic gene cluster was recently identified and shown to consist of seven genes SWN in *Metarhizium robertsii* [24]. The *swnK* gene encodes a multifunctional protein consisted of five domains (A, T, KS, AT, and SDR); inactivation of KS resulted in no detectable swainsonine, demonstrating that this gene is required for synthesis of the toxin. The KS (ketide synthase) domain amongst swainsonine-producing fungi was compared and it was found that *swnK*-KS sequences differed slightly between plant pathogen and non-pathogens but was highly conserved among all swainsonine-producing *Alternaria* spp. [12]. The thioester reductase (TR) component of the SDR domain has not been compared among swainsonine producers. However, Creamer et al. [30] showed high conservation of the PKS (polyketide synthase), NRPS (nonribosomal peptide synthase), and PKS/NRPS genes in the genus *Alternaria* and order Pleosporales through phylogenetic analysis.

*Astragalus pubentissimus* (12A), *A. pubentissimus* (24/2-3), *A. wetherilli* (50-1), *Swainsona canescens*, *S. galegifolia*, *S. luteola*, and *S. brachycarpa* have been shown previously to contain swainsonine (Table S1). The same plants were shown to contain fungal endophytes through isolation from the plants and PCR (polymerase chain reaction) of the fungal ITS region [15,31,32,33]. Noor et al. [15] tested several of the fungi using ITS, *gpd*, and *swnK*-KS primers. Thus, the plants were shown to contain swainsonine and their fungal endophytes were shown to contain the *swnK* gene, which was demonstrated to be essential for swainsonine biosynthesis [15,31,32,33,34]. In addition, the fungal endophyte isolated from *Swainsona canescens* was previously demonstrated to produce swainsonine [17]. The objective of this study was to characterize and determine the taxonomic placement of these fungi through examination of growth habits and morphology of fungal mycelia and conidia as well as phylogenetic comparisons of sequences from the ITS, *swnK-KS* and *swnK-TR* regions.

## 2. Materials and Methods

### 2.1. Plant Collections

*Astragalus* species were collected from the United States, and *Swainsona* species were collected from Australia (Table S1). *Astragalus wetherilli* (50-1) from Garfield, Colorado, *Astragalus pubentissimus* (12A) from Uintah, Utah, and *Astragalus pubentissimus* (24/2-3) from Green River, Wyoming [26]. Fungi were isolated from multiple pressed dried *Astragalus* plant materials stored at New Mexico State University Center for Natural History Collections, Las Cruces, NM, and dried *Swainsona* plant material and seeds (voucher specimen Western Australian Herbarium, Perth) and *Astragalus* were obtained from the Poisonous Plant Research Laboratory, US Department of Agriculture, Logan, Utah. All dried plant materials were stored at room temperature in envelopes. All the plants were previously confirmed for swainsonine presence (Table S1).

### 2.2. Cultural and Morphological Characterization

To isolate fungi from dried plant materials, two pieces of 1 cm long stems, and two pieces of leaflets were surface sterilized as follows: 70% ethanol for 30 s, 20% bleach for 3 min, and rinsed in sterile water for 30 s, then placed on sterile paper towel to dry. The samples were incubated on water agar (WA) media at room temperature [26]. For fungal isolation from *Swainsona* spp., seeds were scarified with fine grit sandpaper, and soaked in sterile water for 30 min. Seeds were then surface sterilized following the previous steps and incubated on WA. Fungi that grew from the plant material were transferred onto acidified potato dextrose agar (APDA) with the same conditions and examined weekly. At least ten fungi isolated from each plant and growing on APDA were observed microscopically using a model LED 5000 RL white light (M165FC stereofluorescence microscope SFM, Leica Microsystems, Buffalo Grove, IL, USA and Exton, PA, USA).

For sporulation examination, isolates were cultured on PDA and potato carrot agar (PCA) [35]. Cultures were incubated in clear plastic boxes beneath white/cool fluorescent light at 25.5 °C for 3–6 weeks [21,25]. Following incubation, isolates were examined for colony morphology, presence of conidia, and morphological characteristics of the conidia. Fungal samples were taken from the center of the colony and the margins, mounted on a microscope slide, and observed with a Nikon microscope (Nikon eclipse E400 Tokyo, Japan) using 20X lenses. Mycelial width and, if present, conidium length and width measurements were taken from 15 randomly selected mycelia and conidia, and mean values and modes were calculated. The number of septa per conidium was also determined for 15 randomly selected conidia.

### 2.3. DNA Extraction, PCR Amplification, and Sequencing

DNA was extracted from fungal cultures using fungal DNA extraction kit (E.N.Z.A. Omega Bio-tek, Inc. Norcross, GA, USA). DNA quality (yield and ratio) for each sample was assessed using a Nanophotometer (IMPLEN P-class S/N:5656) but generally were 10–100 ng/µL. Samples were stored at −20 °C until use. ITS [36] and *swnK*-KS [15] primers were utilized for PCR. swnK-TR primers (9402F 5′-GGAACGCATGATCAGAACGC-3′) and (10211R 5-GCTGCATATTCAAGTGCCCG-3′) were used to amplify a portion of the *swnK* thioester reductase domain. PCR mixture total reaction volume was 50 µL, consisting of 5 µL of 10X standard *Taq* reaction buffer (New England BioLabs^®^ Inc., Ipswich, MA, USA), 1 µL each of 10 mM dNTPs, forward and reverse primers (synthesized by Eurofins Scientific, Luxembourg), 0.25 µL of Taq polymerase (New England BioLabs^®^ Inc.), and 5 µL DNA [25].

PCR samples were then separated by electrophoresis on a 1% agarose gel at 100 V for 45 min and then visualized under UV light. Sizes of PCR amplicons were compared using 100 bp ladders (Promega, Madison, WI, USA). PCR products were purified and sequenced at MCLAB (Molecular Cloning Laboratories, San Francisco, CA, USA).

PCR amplification of the ITS region resulted in a 580–600 bp amplicon, of the *swnK*-KS region resulted in a 740 bp amplicon, and of the *swnK*-TR region resulted in an 810 bp amplicon. Other ITS, *swnK*-KS, and *swnK*-TR sequences from fungi used for comparisons were previously published and GenBank Accession numbers are listed (Table S2). The ITS region and the *swnK*-KS was retested here for all the endophytes because most had been originally determined more than 5 years ago. None of the sequences changed in the retests. Three independent cultures from *Swainsona luteola* were tested since this isolate had not been previously tested. All three gave identical sequences for all regions tested.

### 2.4. Phylogeny

Nucleic acid sequences of ITS and swnK-KS were analyzed using Geneious Prime^®^ 2020.2.2 software and aligned with MUSCLE. Sequences were manually edited to remove any low-quality bases. Maximum parsimony was used to construct phylogenetic DNA trees using PAUP* plugin with the following parameters: heuristic search strategy, fastStep search type and 1000 replications.

Nucleic acid sequences of the swnK-TR domain were analyzed using Geneious Prime^®^ 2025.0.3 software and aligned with MUSCLE 5.1, after which sequences were manually edited to remove any low-quality bases. Trees were produced, selecting Jukes–Cantor as the genetic distance model, and neighbor-joining as a tree build method with no outgroup. The bootstrap option was selected as the resampling method with 1000 replicates and 50% threshold support.

## 3. Results

### 3.1. Cultural and Morphological Characteristics

All fungi isolated from *Astragalus* and *Swainsona* species grew very slowly on all media-tested WA, APDA, and PCA. These endophytes required more than 30–40 days to attain a colony diameter of at least 2 cm. Colony color and texture varied between species, ranging from dark olive green to beige for endophytes isolated from *Astragalus* spp., and light olive green to tan/bronze and gray for *Swainsona* endophytes (Table S3).

Mycelia septate were often arched or wavy, 2–9 μm wide. Two types of hyphae were generally produced: (1) hyphae at the center of the colony, which were highly condensed and produced torulose cells, (2) hyphae at the colony margins, which were less dense than hyphae at the center of the colony. Abundant conidiophores and conidia were observed only with endophytes isolated from *Astragalus pubentissimus* 24/2-3, *Swainsona canescens*, and *Swainsona galegifolia*, while conidia from the endophyte isolated from *A. pubentissimus* 12A were sparse. Conidia were ovate to obclavate to long ellipsoid, straight to inequilateral, single, transseptate; septa thick, dark and rigid, and formed abundant germ tubes, which were wavy or undulate until branching.

1. *Alternaria wetherii*

Collection location

Isolate 50-1 from *Astragalus wetherilli* Garfield Co., Garfield, CO, USA. (D. Cook field collection).

Description

**Figure 1:**
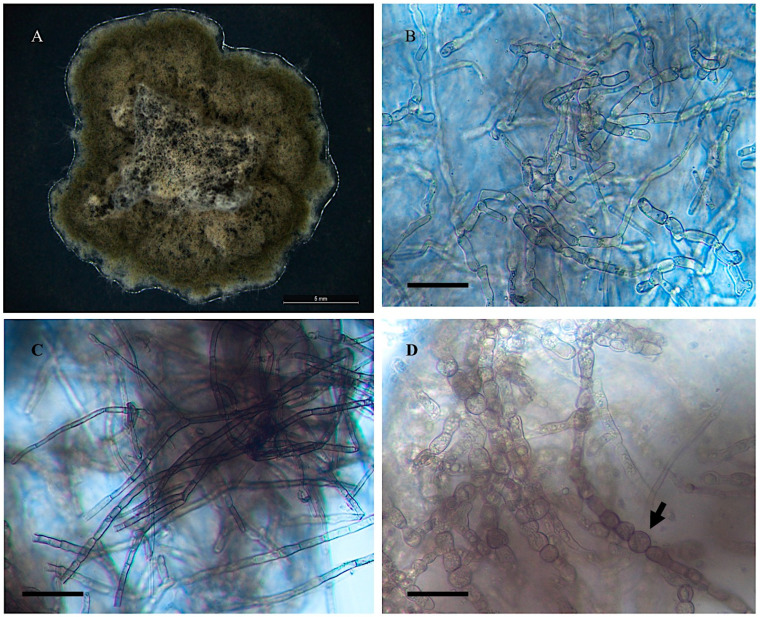
Light microscope images of *Alternaria wetherii* from *Astragalus wetherleii* 50-1. (**A**) Growth of isolate on PDA and room temperature after 25 days. Scale bar = 5 mm. Two types of mycelia: (**B**) torulose cells and (**C**) L = typical linear fungal filaments. (**D**) Chlamydospore indicated by the arrow. Scale bar = 30 μm.

Colonies on PDA were dark olive green with or without light green margin of colony. Mycelial growth was slow, with 0.2–0.8 mm on PDA after 25 days. Densely packed mycelia formed a connected hills-like structure in the colony center and, under the microscope, formed torulose cells. Linear aerial septate mycelia formed at the colony margin, and presence of chlamydospores was observed with this fungal isolate. Mean mycelial had a width of 6.4 μm. Conidia production was not present in this isolate on any media (Figure 1). Nucleic acid sequences are available in GenBank for ITS (MN313519), *gpd* (KM457074), and *swnK*-KS (MN450743) (Table S2).

2. *Alternaria pubentissima*

Collection location

Isolate 12A from *Astragalus pubentissimus* Uintah, Utah, USA (M. Ralph field collection).

Description

**Figure 2:**
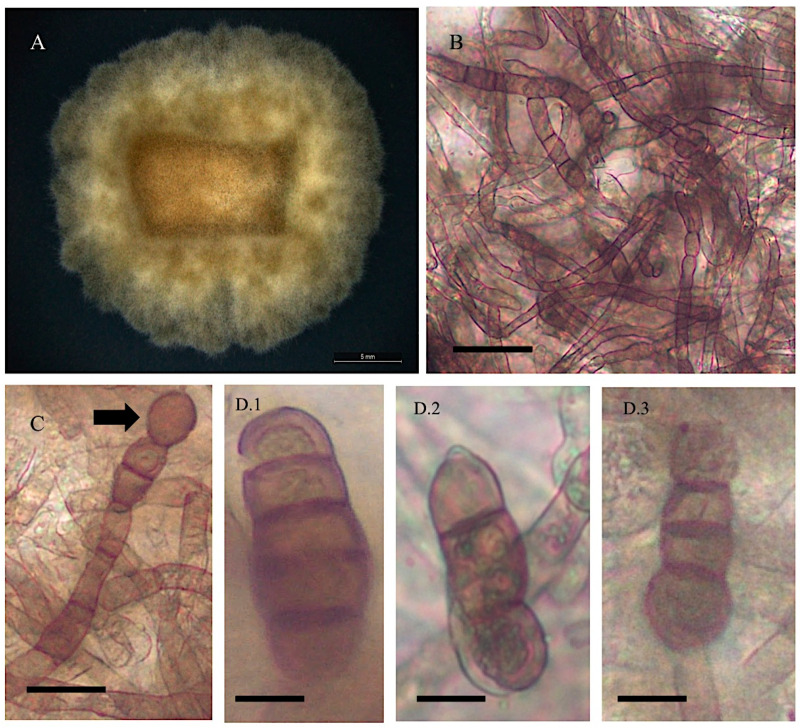
Light microscope images of *Alternaria pubentissima* from *Astragalus pubentissimus* 12A. (**A**) Growth of isolate on PDA and room temperature after 25 days. Scale bar = 5 mm. (**B**) Intertwined hyphal segments. Scale bar = 30 μm. (**C**) Chlamydospore at the end of the hyphal tip indicated by the arrow. Scale bar = 25 μm. (**D.1**–**D.3**) Different shapes of conidium with 2–4 septa. Scale bar = 5 μm.

Colonies on PDA were light green to light brown and tan. The color remained light and the margin turned dark brown with time. Mycelial growth was slow, with 0.3–0.9 mm on PDA after 25 days. Mycelia was not dense compared to other *Astragalus* endophytes in this study and remained flat in culture. Mean mycelial had a width of 5.5 μm. Conidia production was minimal and only a few solitary conidia were produced. The conidia were cylindrical, usually narrowed at the apex, widest right above basal septum, and showed rigid dark 1–4 septa (mean = 2.5, mode = 2). Mean cell length was 56.4 μm, and mean width was 14 μm (Figure 2). Nucleic acid sequences are available in GenBank for ITS (HM588125), *gpd* (MN 326117), and *swnK*-KS (MN450745) (Table S2).

3. *Alternaria pubentissimoides*

Collection location

Isolate 24/2-3 from *Astragalus pubentissimus* Green River, Wyoming, USA. (M. Ralph field collection).

Description

**Figure 3:**
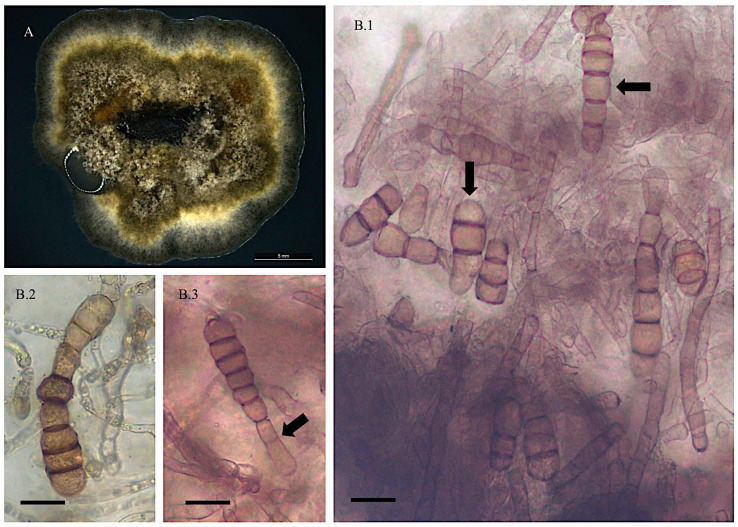
Light microscope images of *Alternaria pubentissimoides* from *Astragalus pubentissimus* 24/2-3. (**A**) Growth of isolate on PDA and room temperature after 25 days. Scale bar = 5 mm. (**B.1**) Abundant conidial formation on PDA and PCA in 25 °C and fluorescent lights after 30 days. (**B.2**.) Conidia formation with 2–3 septa. (**B.3**) Conidium with 5 septa. Scale bar = 10 μm. Arrows indicate conidia was different numbers of septa.

Colonies on PDA were dark olive green. A frequent bright beige/yellow margin around the colony formation was present. Mycelial growth was very slow, with 0.1–0.4 mm on PDA after 25 days. Mycelia was not dense and remained flat in culture. Mean mycelial width was 5.9 μm. Abundant conidia production and wavy germ tubes were observed. Conidia was oval and narrowed at basal cell, and thick dark 1–6 septa (mean = 3.6, mode = 4). Mean cell length was 86.4 μm, and mean width was 15.6 μm (Figure 3). Nucleic acid sequences are available in GenBank for ITS (HM588124), *gpd* (MN 326118), and *swnK*-KS (MN450744) (Table S2).

4. *Alternaria swainsonii*

Collection location

Isolate from *Swainsona canescens* Australia.

Description

**Figure 4:**
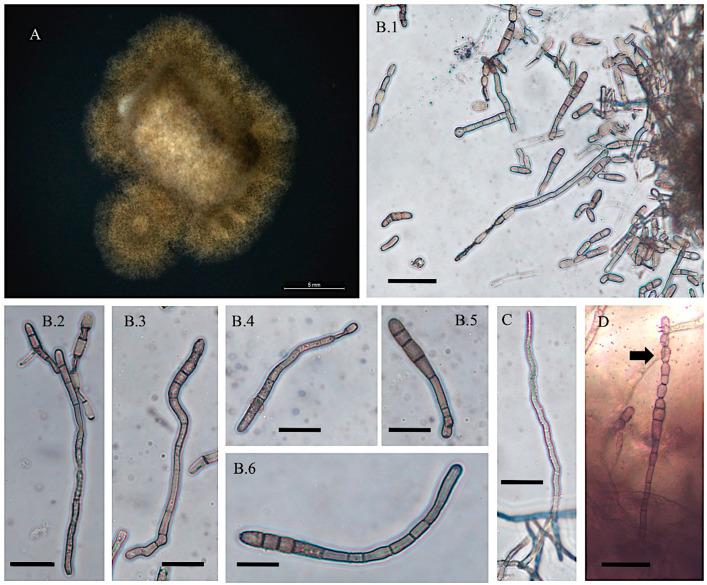
Light microscope images of *Alternaria swainsonii* from *Swainsona canescens*. (**A**) Growth of isolate on PDA and room temperature after 25 days. Scale bar = 5 mm. (**B.1**) Abundant conidial formation on PDA and PCA in 25 °C and fluorescent lights after 30 days. Scale bar = 25 μm. (**B.2**–**B.6**) Different shapes and lengths of conidia. (**C**) Wavy germ tube. Scale bar = 10 μm. (**D**) Conidia in chain. Scale bar = 30 μm. Arrow indicates an unusually long condia in a chain.

Colonies on PDA were light olive green to light brown/bronze and tan. Mycelial growth was slow with 0.2–0.6 mm on PDA after 25 days. Mean mycelial width was 5.3 μm. Abundant conidia production and wavy germ tubes were observed. Conidia were long ovals, slightly wider at the apex, baseball bat-like, with thin dark 1–4 septa (mean = 3.1, mode = 3). Mean cell length was 75 μm, and mean width was 9.6 μm (Figure 4). Nucleic acid sequences are available in GenBank for ITS (JX674068), *gpd* (JX684016), and *swnK*-KS (MN450733) (Table S2).

5. *Alternaria swainsonii*

Collection location

Isolate from *Swainsona galegifolia* Australia.

Description

**Figure 5:**
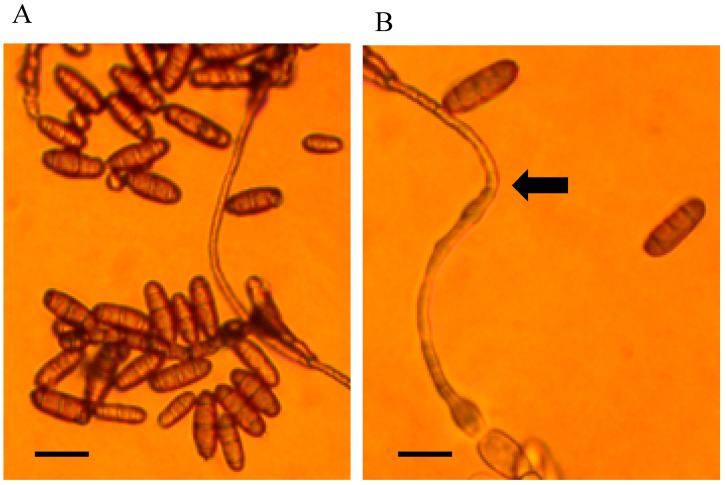
Light microscope images of conidia from endophyte isolated from *Swainsona galegifolia*. (**A**) Conidia in group with 2–3 septa. (**B**) Wavy germ tube indicated by the arrow. Scale bar = 20 μm.

Colonies on PDA were light tan brown. Mycelial growth was slow, with 0.2–0.6 mm on PDA after 25 days. Mean mycelial width was 5.9 μm. Conidia production and wavy germ tubes were observed. Conidia were long ovals, and wider compared to *Alternaria* from *S. canescens*. Thin dark 1–3 septa (mean = 2.1, mode = 3) were observed. Mean cell length was 60.3 μm, and mean width was 12 μm (Figure 5). Nucleic acid sequences are available in GenBank for ITS (JX674068), *gpd* (JX684016), and *swnK*-KS (MN450732) (Table S2).

6. *Alternaria swainsonii*

Collection location

Isolate from *Swainsona luteola* Australia.

Description

**Figure 6:**
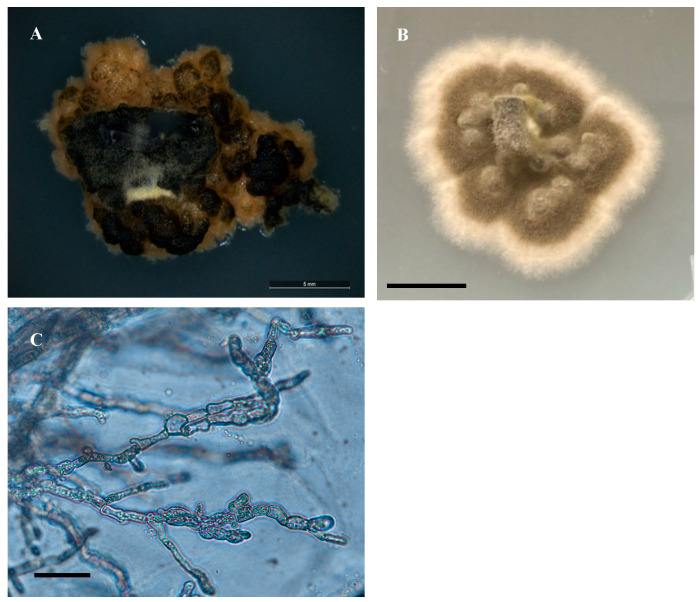
Light microscope images of culture isolated from *Swainsona luteola*. (**A**) Growth of isolate on PDA and room temperature after 15 days. Scale bar = 5 mm. Early growth resembles yeast growth. (**B**) Growth of isolate on PDA at room temperature after 60 days. Scale bar = 10 mm. (**C**) Torulose cells. Scale bar = 30 μm.

Colonies on PDA began as yeast-like and became woolly light olive green with a white margin around colony formation. Mycelial growth was slow, with 0.2–0.4 mm on PDA after 25 days. Mean mycelial width was 5 μm. Formation of torulose cells was observed. No production of conidia on any media was observed (Figure 6).

7. *Alternaria swainsonii*

Collection location

Isolate from *Swainsona brachycarpa* Australia.

Description

**Figure 7:**
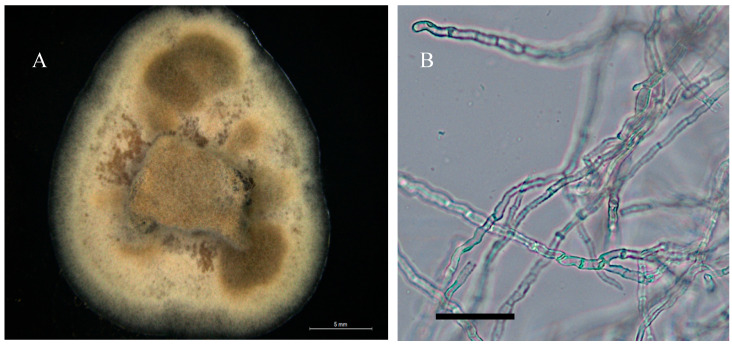
Light microscope images of culture isolated from *Swainsona brachycarpa.* (**A**) Growth of isolate on PDA and at room temperature after 25 days. Scale bar = 5 mm. (**B**) Mycelia. Scale bar = 30 μm.

Colonies on PDA were beige to light gray. Mycelial growth was slow 0.2–0.4 mm on PDA after 25 days. Mean mycelial width was 6.3 μm, and mostly aerial mycelia were present. No production of conidia on any media was observed (Figure 7).

### 3.2. Phylogenetics

**Figure 8:**
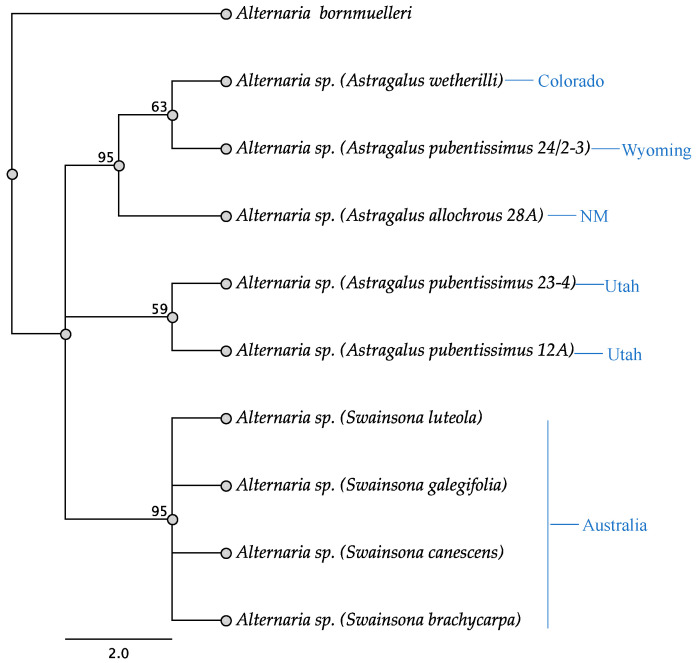
Parsimony tree resulting from analysis of the ITS sequence data. Bootstrap confidence values from 1000 replicates are presented at each node. Location of each plant indicated next to plant name. *Alternaria bornmuelleri* was used as the outgroup.

Sequence analysis of the ITS region showed that the endophytes isolated from *Astragalus* spp. formed two different clades, while *Swainsona* endophytes grouped together into a single clade with 95% bootstrap support (Figure 8). The endophyte from *S. luteola* differed from the other *Swainsona* endophytes by a single base. *Astragalus pubentissimus* 12A endophyte grouped with an endophyte isolated from *A. pubentissimus* 23-4 [15]. In contrast, the endophyte isolated from *A. pubentissimus* 24/2-3 grouped with the *A. wetherilli* 50-1 endophyte and an endophyte from *Astragalus allochrous* [15] (Figure 8).

**Figure 9:**
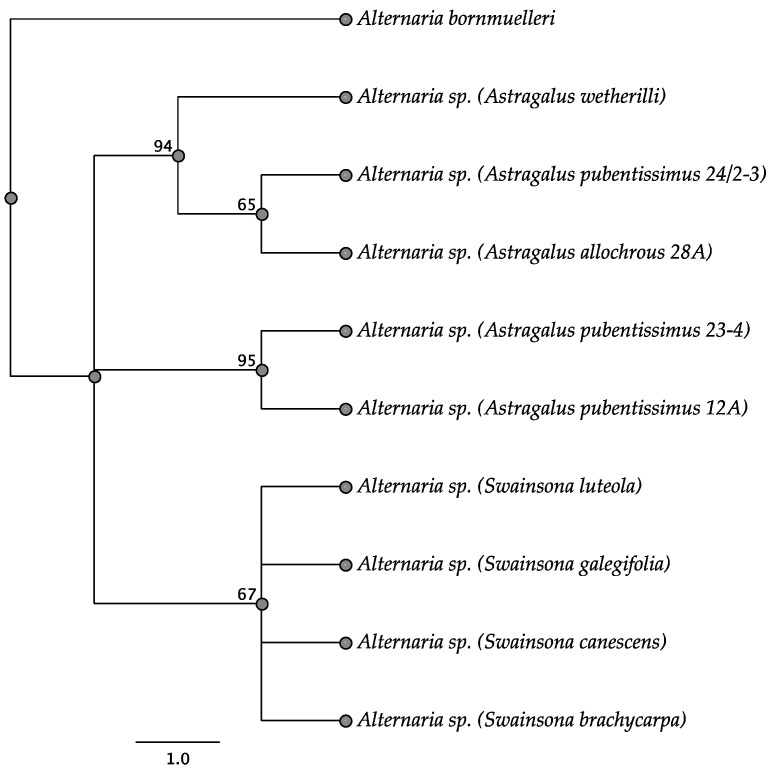
Parsimony tree resulting from analysis of the *swn-KS* sequence data. Bootstrap confidence values from 1000 replicates are presented at each node. Location of each plant indicated next to plant name. *Alternaria bornmuelleri* was used as the outgroup.

The *swnK*-KS tree (Figure 9) showed similar patterns to the ITS tree. *Astragalus wetherilli* 50-1, *A. pubentissimus* 24/2-3, and *A. allochrous* endophytes grouped together, while *A. pubentissimus* 12A and *A. pubentissimus* 23-4 endophytes formed separate clade with 95% bootstraps values. Endophytes isolated from *Swainsona galegifolia* differed by a single base from the other *Swainsona* endophytes.

**Figure 10:**
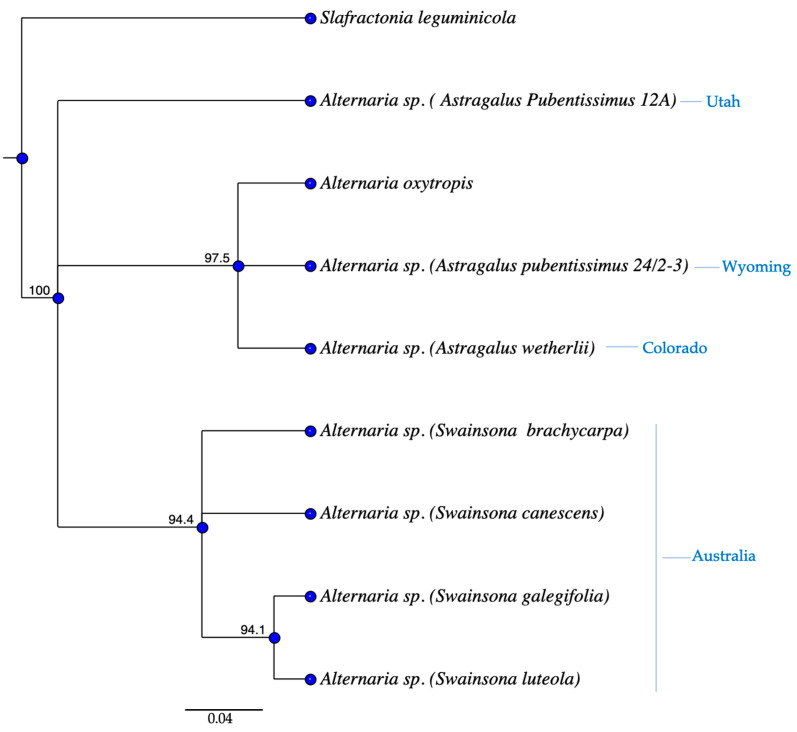
Neighbor joining tree resulting from analysis of the *swn-TR* sequence data. Bootstrap confidence values from 1000 replicates are presented at each node. Location of each plant indicated next to plant name.

The sequence of the *swnH2*–*swnKS* intergenic region (Figure 10) showed very similar patterns as that for *swnKS*. Endophytes from *Astragalus wetherilli* 50-1 and *A. pubentissimus* 24/2-3 grouped together and were similar to *Alternaria oxytropis*, while the *A. pubentissimus* 12A endophyte was distinct. The *Swainsona* endophytes showed 100% identity for the intergenic region. *Slafractonia leguminicola* was distinct from all other fungi.

## 4. Discussion

This study describes the morphological and molecular characters of endophytic fungi isolated from locoweeds *Astragalus* spp. from the western United States and *Swainsona* spp. from Australia. These features were compared with those of previously described *Alternaria* section *Undifilum* species. The new species described here are *Alternaria wetherii*, *Alternaria pubentissima*, and *Alternaria pubentissimoides* from the USA, as well as *Alternaria swainsonii* from Australia. *Alternaria swainsonii* from *Swainsonina canescens* had previously been demonstrated to produce swainsonine [17].

Morphological identification revealed production of ovate to long ellipsoid with occasionally one or two cells distinctly swollen, dark septa, and conidia that produced a wavy germ tube that was undulating until branching; these are distinctive features of *Alternaria* section *Undifilum* spp. [13,17,19,20,25]. Conidia and wavy germ tubes were recorded for *Alternaria pubentissima*, *Alternaria pubentissimoides*, and *Alternaria swainsonii* from *S. canescens* and *S. galegifolium*.

All endophytes tested in this study expressed extremely slow growth. These endophytes varied by the host. *Astragalus* endophytes differed in color and in sporulation from *Swainsona* endophytes, where they grew dark in media with solid masses of mycelium. *Swainsona* endophytes were generally light brown to tan on PDA, which is consistent with previous descriptions [13,17,19,20,25].

The endophytes *Alternaria wetherii*, *Alternaria pubentissima*, and *Alternaria pubentissimoides* from western United States differed in their growth habits, morphology, and phylogeny. The endophytes from *Swainsona* from Australia differed in colony color and condium shape. Conidia from the endophyte isolated from *S. canescens* were longer and thinner compared to those from *S. galegifolia*.

Morphological and molecular differences between endophytes isolated from the same species of plant were observed between *Alternaria pubentissimoides* from Wyoming and *Alternaria pubentissima* from Utah. These endophytes differed in colony color and texture, width and length of conidia, and number of septa.

The swainsonine content of locoweed populations was correlated with endophyte infection [13,28,34]. Sixteen populations of locoweed were analyzed for their swainsonine content and high and low levels of swainsonine in plants were highly correlated with endophyte [28] presence or absence, respectively. Variations in swainsonine levels within plant populations may be due to the presence of different species of endophytes within plant populations.

The molecular markers used in this study were sufficient to differentiate among *Alternaria* species, which is consistent with previous studies [15,19,25,30]. In the ITS, *swnK*-KS, and *swnK*-TR trees, endophytes from different species of *Astragalus* fall into two different clades, which was somewhat expected since they plants were collected from different areas.

In contrast, the fungi isolated from *Swainsona* species were sorted into the same clade using all three genetic markers. For this work, endophytes were re-isolated from all plants and re-sequenced to verify previous results. The species *A. swainsonii* is morphologically diverse, while genetically very similar. These fungi were also distinct from the *Alternaria oxytropis* sequences from the US and China. The *Swainsona*-derived fungi had nearly identical sequences for *swnK*-KS, which is for a coding region that is generally highly conserved [15], as well as for ITS, which is for a non-coded region that is often divergent between fungal species. Including additional genetic markers would make separation of the fungi unlikely since the commonly used markers are also for coding regions that are more conserved than ITS. It is possible that the fungi isolated from *Swainsona* could be sorted into *formae speciales* using whole-genome sequencing, which has been possible for some *Alternaria* isolates [37].

This was the first use of the sequence for the *swnK-TR* domain for phylogenetic comparison. The partial thioester reductase domain was tested for phylogenetic comparisons because it was likely to be specific to swainsonine-producing fungi, but not as highly conserved as the ketide synthase gene. *swnK*-TR differentiated between endophytes isolated from *Astragalus* and *Oxytropis* sp. from those isolated from *Swainsona*. It was identical among all the *Swainsona* endophytes. It provided similar results to that of *swnK*-KS. The *swnK*-TR region is highly conserved, sharing approx. 75% identity with known swainsonine producers *Metarhizium robertsii* and Chaetothyriales endophyte from *Ipomoea carnea*.

In summary, the endophytic nature of *Alternaria* section *Undifilum* species is distinct, as is the production of the toxic alkaloid swainsonine. This unique ecology is a notable character for this group. For example, *Alternaria bornmuelleri* is a leaf parasite that sporulates on its host, spread by airborne conidia, and produces insufficient amounts of swainsonine. In contrast, *Alternaria* section *Undifilum* spp. from the Americas are endosymbionts, seed transmitted, cause no plant pathology, and produce greater amounts of swainsonine. This correlation between swainsonine production and absence of pathology may suggest correlation between swainsonine and symbiosis.

There are many plant endophytic fungi found in diverse locations that produce bioactive compounds [38]. Those compounds and the fungi that produce them are particularly important in agriculture and medicine. Characterization of those fungi, including morphological, molecular, toxicological, and ecological studies will advance knowledge on understanding the relationships between morphology, ecology, and the production of bioactive compounds.

## 5. Conclusions

We have characterized new *Alternaria* section *Undifilum* species isolated from *Astragalus* spp. and *Swainsona* spp. plants. Characterization was based on morphology of the cultured fungus, including spore size and shape and sequence of coding regions (*swnK-KS* and *swnK*-TR) and a noncoding region (ITS). This work also demonstrated greater diversity among the *Alternaria* endophytes isolated from *Astragalus* locoweeds compared to *Swainsona* species. The use of the novel marker *swnK*-TR helped reinforce the higher diversity among *Astragalus* endophytes compared to those from *Swainsona*.

## Data Availability

The original contributions presented in this study are included in the article/Supplementary Material. Further inquiries can be directed to the corresponding author.

## Supplementary Materials

The following supporting information can be downloaded at: https://www.mdpi.com/article/10.3390/jof11070541/s1, Table S1: Plant source and collection location for fungal isolates used for characterization and plant swansonine status. Table S2: Genbank Accession numbers for fungal sequences. Table S3: Morphological Characteristics for Isolated Fungi.
