# Supplementary material for: Morphological and Phylogenetic Characterization of Alternaria Section Undifilum Fungal Endophytes from Astragalus and Swainsona spp
Source: J Fungi (Basel). 2025 Jul 19;11(7):541. doi: 10.3390/jof11070541 (PMC12295656; doi:10.3390/jof11070541)
Supplement: Supplementary file 1 [file jof-11-00541-s001.zip › jof-3683614-supplementary.pdf]

Supplementary Table S1

Plant source and collection location for fungal isolates used for characterization and plant swansonine status

	Plant Origin						
Fungus	Genus	Species	Location	Isolate #	Date collected	Collector	Collection ref / swainsonine ref
Alternaria oxytropis	*Oxytropis*	*sericea*	Raft River, WY	25-2	2005	Michael Ralphs	26/26
Alternaria bornmuelleri	*Securigera*	*varia*	Austria	DAOM 231361	2002	R.A. Shoemaker	19/ND
Alternaria sp.	*Astragalus*	*allochrous*	UT	28A			
***Alternaria* sp.**	***Astragalus***	***wetherilli***	**Garfield, CO**	**50-1**	**2015**	**Daniel Cook**	**32/32**
Alternaria sp.	***Swainsona***	***canescens***	**Australia***	**60S-1**	**2010**		**34/33/23**
	Swainsona	***galegifolia***	**Australia***	**61S-1**	**2010**		**34/33**
	Swainsona	***brachycarpa***	**Australia**	**62S-1**	**2010**	**Daniel Cook**	
	Swainsona	***luteola***	**Australia**	**63S-1**	**2010**	**Daniel Cook**	
***Alternaria* sp.**	***Astragalus***	***pubentissimus***	**Uintah, UT**	**12A**	**2007**	**Michael Ralphs**	**31/31**
			**Green River, WY**	**24-2/3/5**	**2005**	**Michael Ralphs**	**26/26**
			UT	23/4	2005	Michael Ralphs	26/26

Bold indicates plants and fungi characterized in this work.

Collection ref/ swainsonine ref indicates the reference that detailed the plant collection/the setection of swainsonine in the plant/the detection of swainsonine in the cultured fungus, ND=not detected, * = Plant materials were purchased from a French company, but originated in Australia.

Supplementary Table S2

Genbank Accession numbers for fungal sequences

	Plant Origin				swnK-TR	swnK-KS	ITS	GPD
Fungus	Genus	Species	Location	Isolate #	Genbank accession #	Genbank accession #	Genbank accession #	Genbank accession #
Alternaria oxytropis	*Oxytropis*	*sericea*	WY	25-2	KY365741.1	MN450736	HM588133	JN632558
Alternaria bornmuelleri			Austria	DAOM 231361		MN450758	FJ357317	FJ357305
Alternaria sp.	*Astragalus*	*allochrous*	UT	28A		MN450759	MN313510	MN326116
Alternaria wetherii	*Astragalus*	*wetherilli*	CO	50-1	**PV290889**	MN450743	MN313509	KM457074
Alternaria swainsonii	*Swainsona*	*canescens*	Australia	60S-1	**PV290886**	MN450733	JX674068	JX684016
	Swainsona	*galegifolia*	Australia	61S-1	**PV290886**	MN450732	JX674068	JX684016
	Swainsona	*brachycarpa*	Australia	62S-1	**PV290886**	MN450733	JX674068	
	Swainsona	*luteola*	Australia	63S-1	**PV290886**	MN450733	JX674068	
Alternaria pubentissima	*Astragalus*	*pubentissimus*	UT	12A	**PV290887**	MN450745	HM588125	MN326117
Alternaria pubentissimoides			WY	24-2/3/5	**PV290888**	MN450744	HM588124	MN326118
Alternaria sp.			UT	23-4		MN450760	HM588125	
Slafractonia leguminicola			WV	ATCC 26280	KY365746.1	KY365746	KM376910	AB985614

(swnK-TR) Thioester reductase domain of *swn*K

(ITS) internal transcribed spacer

(*GPD*) *glyceraldehyde-3-phosphate dehydrogenase*

(swnK-KS) Ketide Synthase domain of *swn*K

Genbank accession marked in bold represent sequence that was determined in this work.

Supplementary Table S3

Morphological Characteristics for Isolated Fungi

Fungus		Plant			Colony			
Genus	Species	Genus	Species	Isolate #	Color	Mycelia width um	Conidia size um	Septa average
Alternaria	*wetherii*	*Astragalus*	*wertherilli*	50-1	Dark olive green	6.4		
Alternaria	*swainsonii*	*Swainsona*	*canescens*	60S-1	Light green to brown	5.3	75 x 9.6	3.1
	swainsonii	*Swainsona*	*galegifolia*	61S-1	Light tan - brown	5.9	60.3 x 12	2.1
	swainsonii	*Swainsona*	*brachycarpa*	62S-1	Beige to grey	6.3		
	swainsonii	*Swainsona*	*luteola*	63S-1	Light green	5.0		
Alternaria	*pubentissima*	*Astragalus*	*pubentissimus*	12A	Light green to tan	5.5	56.4 x 14	2.5
Alternaria	*pubentissimoides*	*Astragalus*	*pubentissimus*	24-2/3/5	Dark olive green	5.9	86.4 x 15.6	3.6
